# Pulmonary Veno-Occlusive Disease: A Clue to Underlying Lymphoma

**DOI:** 10.7759/cureus.102583

**Published:** 2026-01-29

**Authors:** Chaynez Rachid, Othman Abouobayd, Mohamed Ijim, Hind Rachidi, Hanane Rais, Oussama Fikri, Lamyae Amro

**Affiliations:** 1 Pulmonology, Morpho-Sciences Research Laboratory, Faculty of Medicine and Pharmacy of Marrakech, Cadi Ayyad University, Arrazi Hospital, University Hospital Center Mohammed VI, Marrakech, MAR; 2 Pathology and Laboratory Medicine, Mohamed VI University Hospital of Marrakech, Marrakech, MAR; 3 Pathology, Morpho-Sciences Research Laboratory, Faculty of Medicine and Pharmacy of Marrakech, Cadi Ayyad University, Arrazi Hospital, University Hospital Center Mohammed VI, Marrakech, MAR

**Keywords:** diffuse ground-glass opacities, hodgkin lymphoma, lymphadenopathy, post-capillary pulmonary hypertension, pvod

## Abstract

Pulmonary veno-occlusive disease (PVOD) is a rare cause of post-capillary pulmonary hypertension. Unlike the more common pulmonary arterial hypertension (PAH), PVOD specifically involves the small pulmonary veins. While often idiopathic or genetic, PVOD can occasionally be the presenting symptom of an occult malignancy, such as lymphoma, through direct venous infiltration or extrinsic compression. We report the case of a 31-year-old patient who presented with progressive dyspnea, non-productive cough, and signs of right-sided heart failure. Initial thoracic computed tomography (CT) imaging showed characteristic signs of PVOD: diffuse ground-glass opacities, thickened interlobular septa, and mediastinal lymphadenopathy. Although right heart catheterization was indicated to confirm pre-capillary pulmonary hypertension, it could not be carried out because of the patient’s clinical deterioration*. *A subsequent biopsy of the enlarged axillary lymph nodes confirmed the diagnosis of Hodgkin's lymphoma, suggesting that the pulmonary vascular obstruction was a secondary phenomenon. PVOD is a life-threatening condition that requires high clinical suspicion. In patients presenting with radiological features of PVOD and especially lymphadenopathy, a thorough workup for lymphoma is mandatory. Distinguishing between primary PVOD and malignancy-associated PVOD is crucial, as the use of standard pulmonary vasodilators can trigger fatal pulmonary edema in these patients.

## Introduction

Pulmonary veno-occlusive disease (PVOD) represents a rare and devastating subset of pulmonary arterial hypertension, characterized by the progressive fibrous occlusion of small pulmonary veins and capillaries. While the idiopathic form is frequently linked to EIF2AK4 genetic mutations or toxic exposures, PVOD can also emerge as a secondary phenomenon. Among its most elusive triggers is malignant lymphoma, where the disease serves as the inaugural clinical manifestation of an occult hematological malignancy. The pathophysiology of lymphoma-associated PVOD is complex, often involving direct intravascular infiltration by neoplastic cells or extrinsic compression of the pulmonary venous system by mediastinal lymphadenopathy. This clinical overlap creates a significant diagnostic "trap": the symptoms of progressive dyspnea and right heart failure can easily be attributed to more common etiologies, delaying the detection of the underlying lymphoma. Furthermore, recognizing this association is of paramount clinical importance. The standard treatment for pulmonary hypertension - vasodilator therapy - is supported by limited evidence and is associated with notorious side effects in PVOD patients, as it can precipitate life-threatening pulmonary edema. When PVOD is the revealing sign of a lymphoma, the diagnostic window is narrow, and the prognosis often hinges on the rapid initiation of systemic chemotherapy rather than traditional pulmonary vascular targeting. This article presents a clinical case that underscores the necessity of including lymphoma in the differential diagnosis of PVOD, particularly when atypical radiological features or systemic symptoms are present [1].

## Case presentation

**Figure 1:**
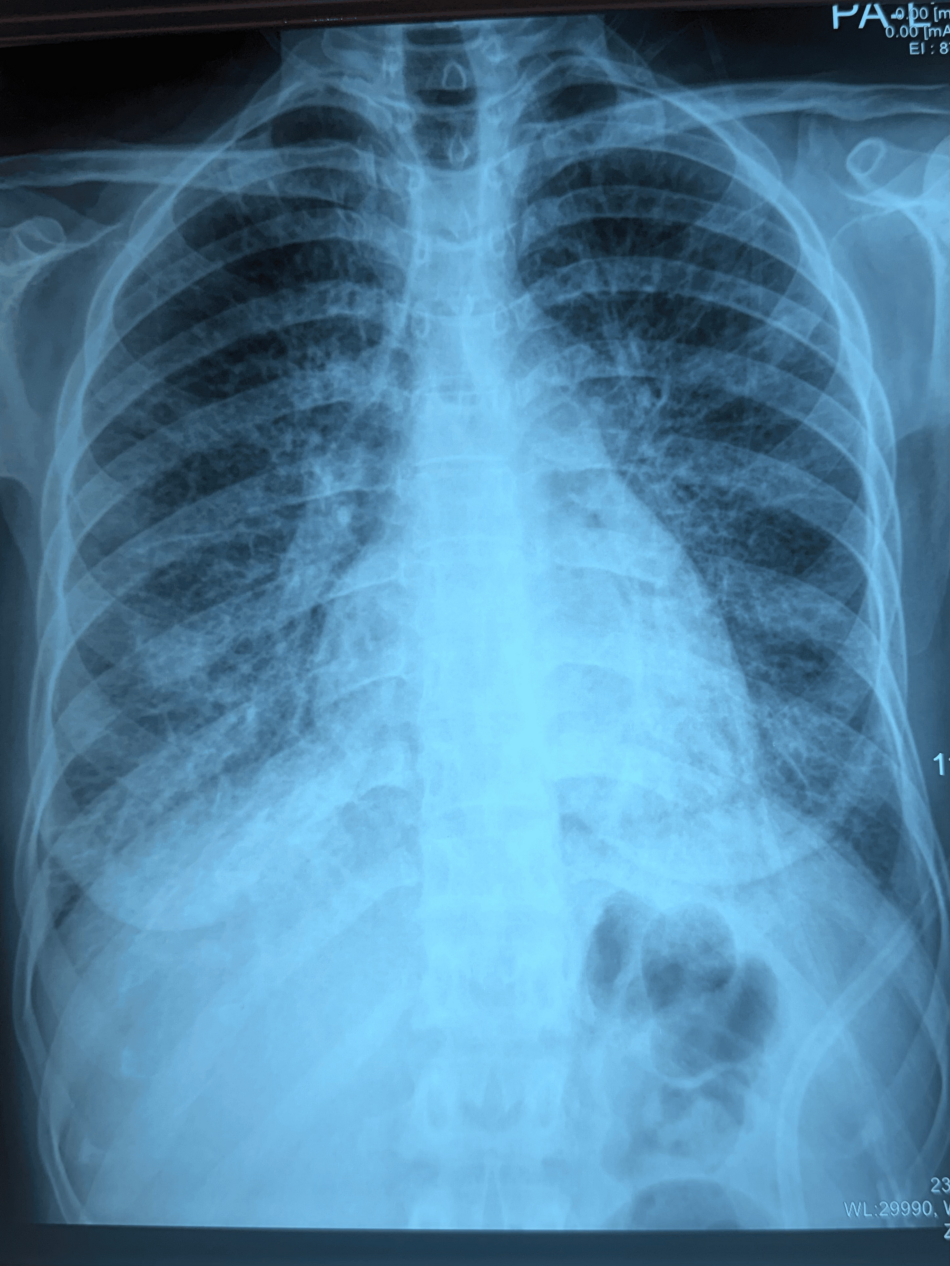
Posteroanterior chest radiograph showing the presence of an interstitial syndrome The image shows diffuse bilateral infiltrates with ground-glass attenuation.

We present the case of a 31-year-old female with no significant history and no professional or environmental exposure. Two years before her admission, she presented with a dry cough, dyspnea on exertion, and polyarthralgia of inflammatory appearance in the context of general deterioration. Clinical examination revealed a conscious patient, tachypneic with a respiratory rate of 24 breaths per minute, with an oxygen saturation of 70% on room air, improving to 94% under 3 L/min of supplemental oxygen. The respiratory examination was unremarkable. Digital clubbing was noted, along with a mobile, painless left axillary lymphadenopathy. The chest x-ray showed an interstitial syndrome with diffuse reticulomicronodular opacities and diffuse bilateral ground-glass opacities (Figure 1).

Sputum microscopy and Xpert MTB/RIF (*Mycobacterium tuberculosis*/rifampicin resistance) assay were negative. The angiotensin-converting enzyme (ACE) level was 61 IU/L (normal range: <70 IU/L), reflecting macrophage activation and the presence of granulomatous reactions associated with lymphoma (sarcoid-like reaction). The serum and urinary calcium-phosphate profile showed a serum calcium level of 100.21 mg/L, urinary calcium level of 269 mg/24 h, serum phosphate level of 46 mg/L, and urinary phosphate level of 2029 mg/24 h (elevated). The corrected serum calcium level was slightly decreased (2.1 mmol/L), consistent with an inflammatory context associated with lymphoma, probably related to hypoalbuminemia (Table 1).

**Table 1:** Laboratory investigations

Parameters	Patient values	Reference range
Serum calcium	100.21 mg/L	85–105 mg/L
Urinary calcium	269 mg/24 h	100–300 mg/24 h
Serum phosphate	46 mg/L	25–45 mg/L
Urinary phosphate	2029 mg/24 h	400–1300 mg/24 h
Corrected serum calcium	2.1 mmol/L	2.20 à 2.60 mmol/L

**Figure 2:**
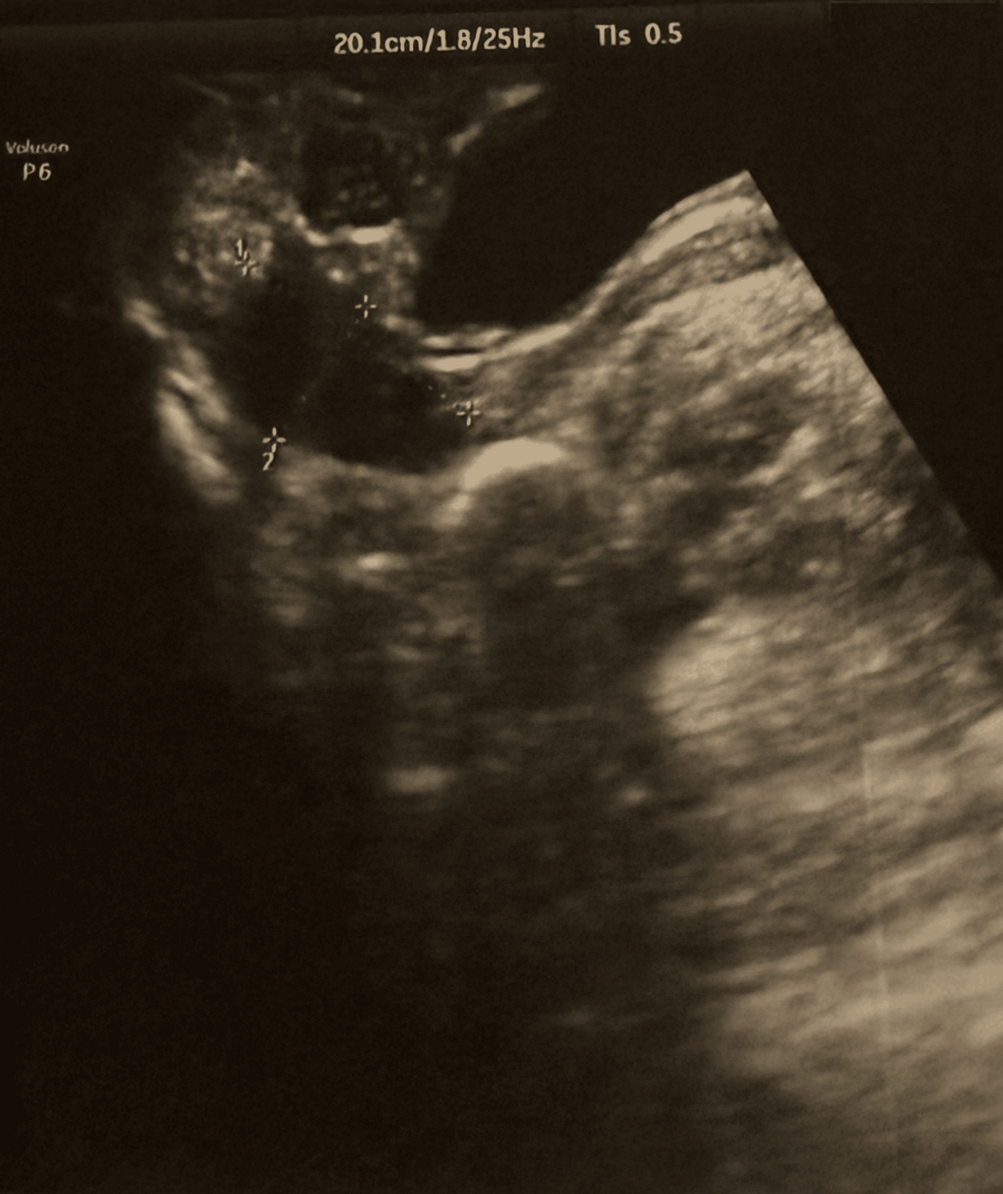
Axillary ultrasound image showing left axillary lymphadenopathy This image shows the left axillary lymphadenopathy measuring 3.8 cm in its long axis, with an oval shape.

The respiratory PCR panel was negative. The search for *Pneumocystis jirovecii* in sputum was negative. Axillary ultrasound showed left axillary lymphadenopathy measuring 3.8 cm (Figure 2).

**Figure 3:**
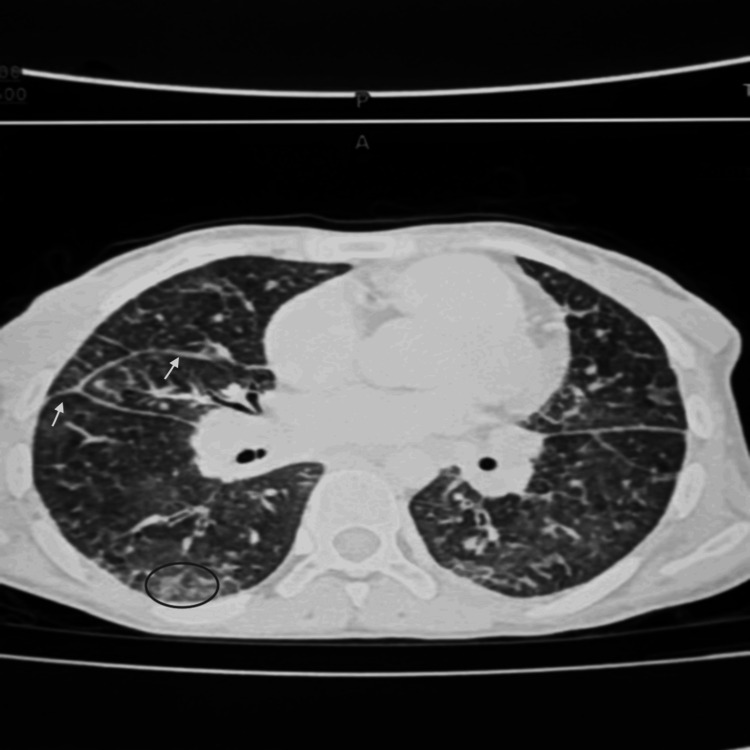
Thoracic CT scan images showing interlobular septal thickening and ground-glass opacities Diffuse bilateral thickening of the interlobular septa, with bilateral areas of ground-glass opacities, more marked in the upper lobes and both basal regions, was observed. Branching centrilobular micronodules and parenchymal nodules were present. Mediastinal lymphadenopathy involving the subcarinal region, para-aortic area, Barety’s space, and bilateral hilar regions was present. Bilateral pleural effusion and right fissural pleural effusion, associated with a mild to moderate pericardial effusion, were observed.

The thoracic computed tomography (CT) scan showed bilateral alveolo-interstitial involvement associated with centrilobular ground-glass nodules and scattered micronodules in both lung fields, thickening of the interlobular septa, as well as mediastinal and left axillary lymphadenopathy. There is also an associated pleural and pericardial effusion. Overall, the suggestion of a crazy-paving pattern is highly suggestive of PVOD (Figure 3).

**Figure 4:**
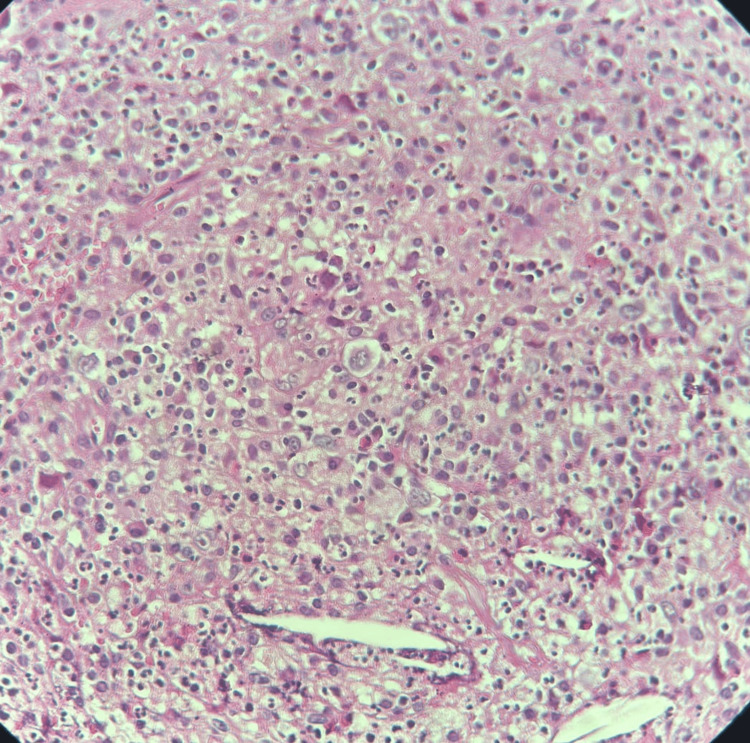
Reed–Sternberg cells and Hodgkin cells The tumor proliferation is composed of large cells, mononucleated or binucleated, with one or two central nucleoli. The cytoplasm is moderately abundant and eosinophilic. This tumor proliferation is found within a granulomatous inflammatory stroma containing a few eosinophilic polymorphonuclear cells (H&E stain, ×40 magnification).

Neither bronchoscopy nor lung biopsy could be performed due to the patient’s respiratory condition. A biopsy of the axillary lymph node was performed, which showed that the tumor proliferation was composed of large cells compatible with Reed-Sternberg cells and Hodgkin cells (Figure 4).

**Figure 5:**
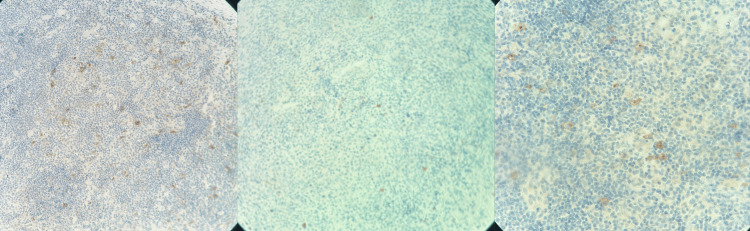
Immunohistochemical analysis demonstrates findings in favor of Hodgkin lymphoma A moderate and diffuse membranous expression of anti-CD15 and anti-CD30 antibodies was observed in the large tumor cells. The large tumor cells lacked expression of anti-CD20, with a positive internal control (H&E stain, ×20 magnification).

Immunohistochemical analysis demonstrates findings in favor of Hodgkin lymphoma (Figure 5).

Arterial blood gas analysis showed a pH of 7.46, PaO_2_ of 32.2 mmHg, PaCO_2_ of 33.8 mmHg, and HCO_3_^-^ of 23.9 mmol/L, with an oxygen saturation of 76%, consistent with respiratory alkalosis associated with severe hypoxemia (Table 2).

**Table 2:** Arterial blood gas results This table shows the Shunt effect characterized by severe hypoxemia with hypocapnia.

Parameters	Patient values	Reference range
PH	7.46	7.35–7.45
PaO_2_	32.2 mmHg	80-100 mmHg
PcO_2_	33.8 mmHg	35–45 mmHg
HCO_3_^-^	24.9 mmol/L	22–26 mmol/L
S0_2_	76%	95%–100%

**Figure 6:**
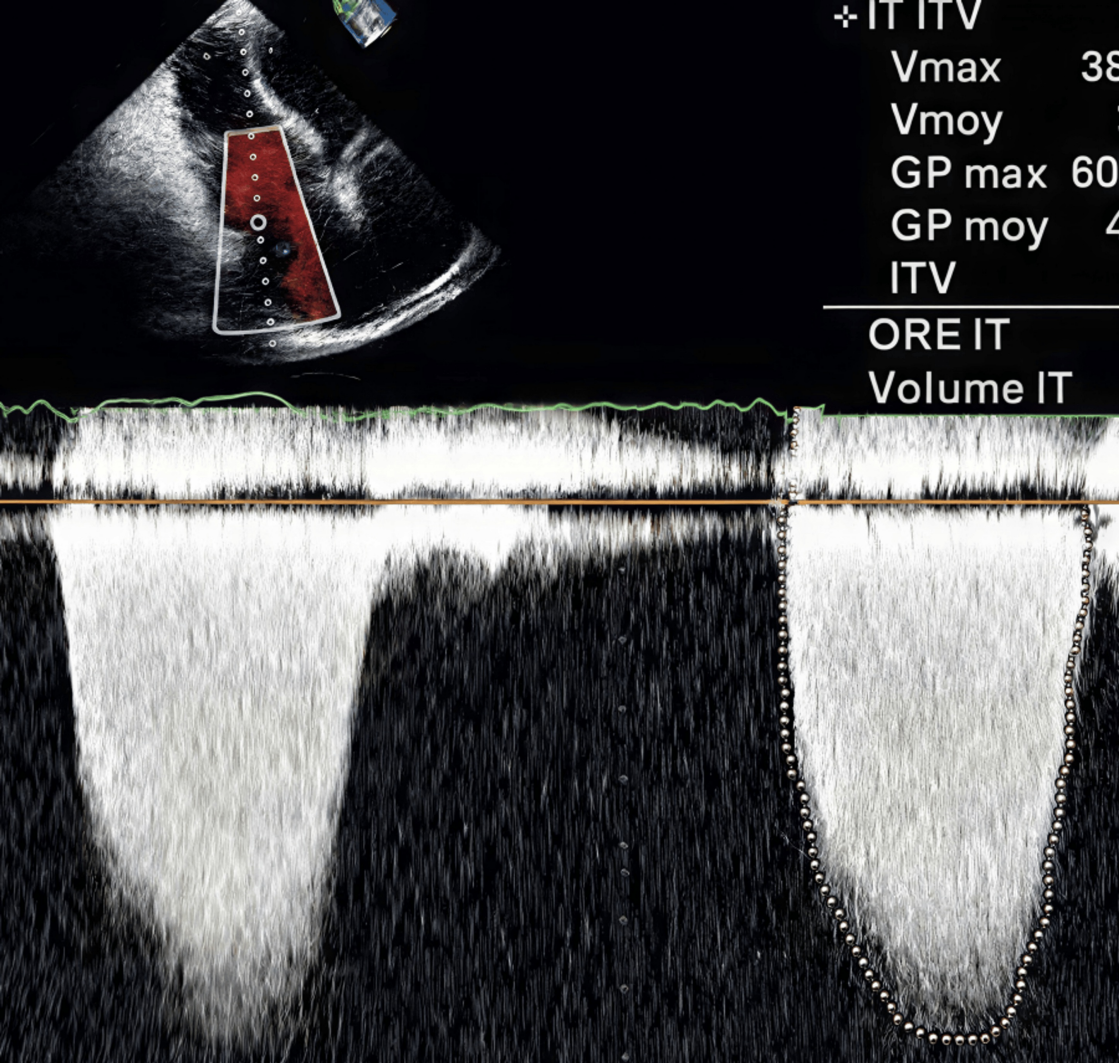
Transthoracic echocardiogram image with pulmonary hypertension This image shows a dilated and hypertrophied right ventricle, moderate mitral regurgitation, and pulmonary hypertension estimated at 70 mmHg.

Transthoracic echocardiography showed a dilated and hypertrophied right ventricle, no pericardial effusion, and pulmonary hypertension estimated at 70 mmHg (Figure 6). Right heart catheterization could not be performed due to very severe pulmonary hypertension with a high risk of hemodynamic decompensation.

The patient received standard first-line chemotherapy for Hodgkin lymphoma with appropriate supportive care. The total duration of the treatment was 12 months, with regular follow-up conducted once per month. A favorable clinical and radiological response was observed, leading to complete remission at the end of treatment. Therapy was well tolerated, with only mild and transient adverse effects. The patient remains disease-free under regular follow-up.

## Discussion

PVOD is a rare and often underdiagnosed cause of pulmonary hypertension, characterized by progressive fibrotic occlusion of small pulmonary veins and venules. Clinically, PVOD closely mimics pulmonary arterial hypertension (PAH), yet its prognosis, management, and underlying mechanisms differ substantially. The present case highlights PVOD as an unusual but critical manifestation revealing an underlying lymphoma, emphasizing the need to consider secondary and potentially reversible causes in patients presenting with rapidly progressive pulmonary hypertension [2].

PVOD may occur in idiopathic, heritable, drug-induced, or secondary forms. Secondary PVOD has been described in association with connective tissue diseases, infections, exposure to chemotherapeutic agents, and malignancies, particularly hematologic cancers. Lymphoma-associated PVOD remains exceptionally rare, and its pathophysiology is not fully elucidated. Proposed mechanisms include direct venous infiltration by malignant cells, paraneoplastic immune-mediated endothelial injury, cytokine-driven fibroproliferative remodeling, or microthrombotic processes affecting the post-capillary pulmonary vasculature [3].

The diagnostic challenge of PVOD lies in its clinical and hemodynamic resemblance to PAH. However, several features should raise suspicion for PVOD, including profound hypoxemia, severely reduced diffusing capacity for carbon monoxide (DLCO), radiological findings such as centrilobular ground-glass opacities, interlobular septal thickening, and mediastinal lymphadenopathy. In the context of lymphoma, these imaging features may be mistakenly attributed to infection, pulmonary edema, or lymphangitic carcinomatosis, potentially delaying the correct diagnosis [4].

Importantly, the recognition of PVOD has major therapeutic implications. Unlike PAH, the use of pulmonary vasodilators in PVOD may precipitate life-threatening pulmonary edema due to increased capillary hydrostatic pressure in the setting of venous obstruction. In secondary PVOD related to malignancy, management should primarily focus on treating the underlying disease. Several reports suggest that effective lymphoma therapy may stabilize or partially reverse pulmonary vascular involvement, reinforcing the concept that PVOD in this context may represent a paraneoplastic or reactive process rather than irreversible vascular remodeling [5].

This case underscores the importance of a multidisciplinary approach combining clinical, radiological, hemodynamic, and oncological evaluation. Early identification of lymphoma in patients with suspected PVOD is crucial, as timely initiation of disease-specific therapy may significantly impact prognosis. Some lymphoma treatments may worsen PVOD due to the use of specific chemotherapeutic agents employed in its management. Moreover, this association highlights the need to systematically search for occult malignancy when PVOD is diagnosed, particularly in the presence of lymphadenopathy, systemic symptoms, or atypical imaging findings [6].

In conclusion, PVOD can be the initial manifestation of an underlying lymphoma and should prompt an extensive etiological workup. Recognizing this rare association is essential to avoid inappropriate treatment with pulmonary vasodilators and to enable targeted therapy of the causative malignancy. Increased awareness among clinicians may lead to earlier diagnosis, improved management strategies, and potentially better outcomes in this otherwise devastating condition [7].

## Conclusions

PVOD represents a rare and frequently overlooked cause of pulmonary hypertension, with clinical and radiological features that closely resemble PAH. This case highlights PVOD as an unusual presenting manifestation of an underlying lymphoma, underscoring the importance of considering secondary and potentially reversible etiologies. Early recognition of this association is crucial, as misdiagnosis may lead to inappropriate use of pulmonary vasodilators and severe clinical deterioration. A thorough etiological assessment, particularly in the presence of suggestive imaging findings or systemic features, is essential. Identifying and treating the underlying malignancy may significantly influence disease progression and patient outcomes. Increased awareness of lymphoma-associated PVOD may improve diagnostic accuracy and guide optimal multidisciplinary management of this challenging condition.
